# Psychological and physiological evidence for an initial ‘Rough Sketch’ calculation of personal space

**DOI:** 10.1038/s41598-021-99578-1

**Published:** 2021-10-25

**Authors:** Roger B. H. Tootell, Sarah L. Zapetis, Baktash Babadi, Zahra Nasiriavanaki, Dylan E. Hughes, Kim Mueser, Michael Otto, Ed Pace-Schott, Daphne J. Holt

**Affiliations:** 1 Department of Psychiatry, Massachusetts General Hospital, Charlestown, MA USA; 2 Harvard Medical School, Boston, MA USA; 3 Athinoula A. Martinos Center for Biomedical Imaging, Massachusetts General Brigham Hospital, Boston, MA USA; 4 Department of Radiology, Massachusetts General Hospital, 149 13th St, Charlestown, MA USA; 5 Center for Psychiatric Rehabilitation, Boston University, Boston, MA USA; 6 Department of Psychological and Brain Sciences, Boston University, Boston, MA USA

**Keywords:** Computational biology and bioinformatics, Neuroscience, Physiology, Psychology

## Abstract

Personal space has been defined as “the area individuals maintain around themselves into which others cannot intrude without arousing discomfort”. However, the precise relationship between discomfort (or arousal) responses as a function of distance from an observer remains incompletely understood. Also the mechanisms involved in recognizing conspecifics and distinguishing them from other objects within personal space have not been identified. Accordingly, here we measured personal space preferences in response to real humans and human-like avatars (in virtual reality), using well-validated “stop distance” procedures. Based on threshold measurements of personal space, we examined within-subject variations in discomfort-related responses across multiple distances (spanning inside and outside each individual’s personal space boundary), as reflected by psychological (ratings) and physiological (skin conductance) responses to both humans and avatars. We found that the discomfort-by-distance functions for both humans and avatars were closely fit by a power law. These results suggest that the brain computation of visually-defined personal space begins with a ‘rough sketch’ stage, which generates responses to a broad range of human-like stimuli, in addition to humans. Analogous processing mechanisms may underlie other brain functions which respond similarly to both real and simulated human body parts.

## Introduction

The widespread recent practice of social distancing during the COVID-19 pandemic has greatly influenced the way we position ourselves relative to others. This social distancing has generated renewed interest in interpersonal space regulation^1,2^. Historically, the study of personal space is often traced to ethological observations in the middle twentieth century^3–5^, related to the ‘fight or flight’ interactions between different animals, including predator and prey species. Subsequent studies clarified that the more specific behavior of personal space regulation occurs between members of the same species, i.e. conspecifics^6–9^. Typically, personal space has been studied in humans, but analogous behavioral interactions have been reported between macaque monkeys^10^.

In studies conducted in humans, personal space has been defined as ‘the area individuals maintain around themselves into which others cannot intrude without arousing discomfort’^11,12^. Often in real life, and in laboratory studies of personal space, a subject positions themself at a consistent distance from an unfamiliar person. The size of personal space or interpersonal distance can vary widely across individuals, often averaging between 60 to 100 cm^11^. Reviews of the literature have concluded that personal space is influenced by age, physical and psychological variables, psychological disorders^13–15^, gender^16–20^, and cultural differences. Nevertheless, an individual’s preferred personal space size tends towards a relatively stable default value (a “trait-like” preference), which may be further influenced by these modulating factors.

**Figure 1:**
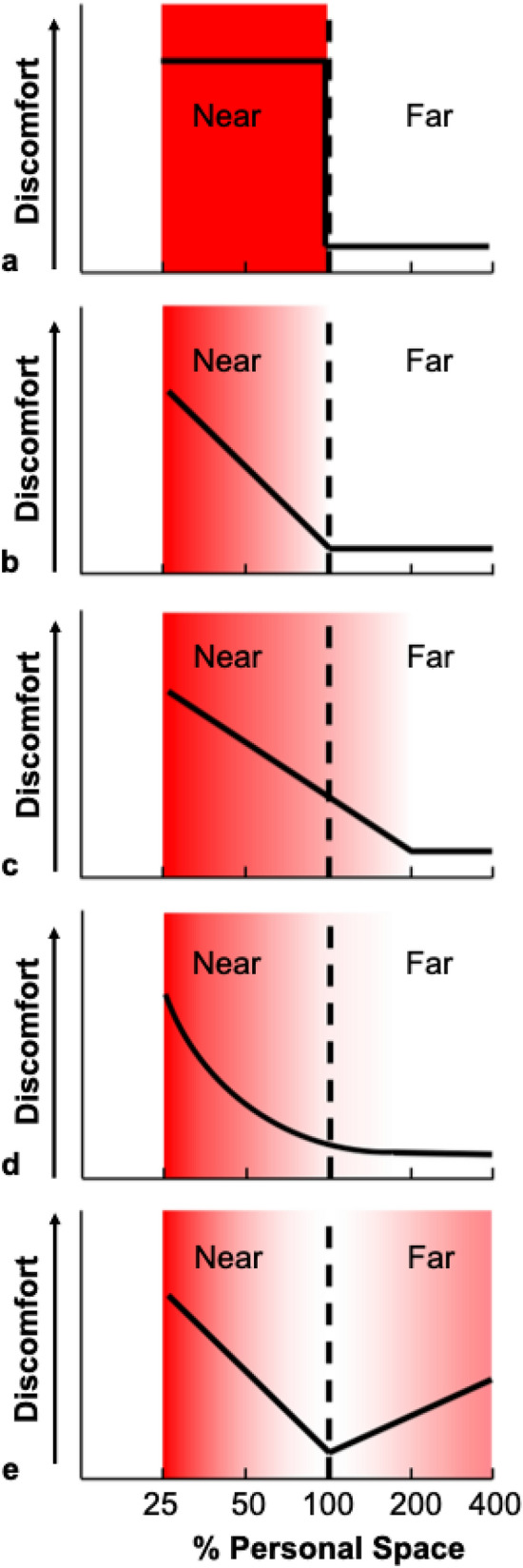
Models of personal space. Qualitative models of variations in discomfort (on the y axis) to an unfamiliar human positioned at different distances from a subject, as a percentage of the subjects’ personal space size (x axis). The dashed vertical line represents the personal space boundary, i.e. 100% of the average SDP value for each subject. The models include a: (**a**) bubble model, (**b**) linear gradient model, (**c**) linear gradient with a threshold, (**d**) power law, and (**e**) social equilibrium model. The panels also show the hypothesized changes in discomfort for each model, as variations in red shading (most discomfort = most saturated red).

Several models of the shape of personal space (as defined by discomfort in response to the proximity of others) are illustrated in Fig. 1. Early models of personal space included a snail shell^21^, or a ‘soap bubble’^22,23^. Those models implied a discrete boundary around the subject, beyond which the level of discomfort due to the presence of another person changes dramatically**,** e.g. as a step function in the discomfort-by-distance relationship (Fig. 1a). Subsequently, proposed models were more graded, including an electromagnetic gradient or a compressible spring^24^ (e.g. Fig. 1b–e). However, the exact form of the personal space gradient has remained difficult to define. Hayduk^24^ suggested that a combination of linear and nonlinear factors determine personal space (Fig. 1d). Other studies proposed that the function of distance-by-discomfort is U- or V-shaped^25,26^, as elaborated in the social equilibrium model (Fig. 1e)^19,27,28^. Another study concluded that the relationship between personal space and absolute distance is ‘curvilinear’^29^. Adding to the inherent challenges in defining this function are the methodological differences among studies, and the limited number of data points used to estimate the shape of the function in several experiments.

Based on this literature, the primary goal of the current study was to clarify the ‘distance-by-discomfort’ function of personal space processing, in ways that are complementary to prior experiments, in several respects. First, prior studies have typically investigated personal space across subjects based on *absolute* physical distance. Here we instead measured responses to distances from the subject after normalization to the threshold personal space measurement in each given individual. As an analogy, heights across human individuals have a certain variability when based on *absolute* distance—but the variability decreases significantly when height is normalized by the size of other body parts, e.g. arm length^30–32^.

Second, we measured the discomfort-by-(normalized)-distance functions within a given individual using both (1) discomfort ratings and (2) measurements of ‘arousal’, based on palmar skin conductance responses (SCR). Such dual measurements within each subject made it possible to test the degree to which self-reported discomfort ratings corresponded to visceral discomfort at correspondingly closer interpersonal distances. More broadly, the dual measurements allowed us to test whether psychological ratings and physiological measurements yielded similar or disparate functions.

Third, these measurements were collected during intrusions into personal space of both humans and human-like avatars. By comparing responses to real and virtual humans, we tested a neural model in which the brain calculates personal space in multiple stages. In this model, the brain first calculates a default ‘rough sketch’ of nearby objects and their spatial arrangement relative to the observer, along with ‘tags’ for objects that *may be* socially or personally relevant. Neurobiological evidence suggests that this stage might be linked with the re-encoding of surrounding space from eye-centered to body- (or person-) centered space, which may involve posterior parietal cortex^33–40^. Subsequent stages involving higher level cortical areas presumably fine-tune decisions about whether a given intruder is in fact human, and account for more subtle modulating factors (e.g. age, culture, gender, psychological characteristics), and then mediate motor and visceral responses to intruders.

Crucially here, the human-like avatars were instantly and unambiguously distinguishable from real humans. Our rough sketch hypothesis predicted that the expected effects of personal space intrusion (e.g. increased subjective discomfort and increased physiological responses at distances that are at or within the individual’s personal space boundary) would be evoked similarly in response to both humans and avatars.

## Methods

### Subjects

Nineteen healthy subjects were included in the main experiment (9 female, mean age = 30.6 ± 11.3 years). Data collected in a previously assessed cohort of 30 healthy subjects (8 female, mean age = 26.1 ± 6.1) were also examined to further validate the reliability and stability of the conventional procedures we used to measure the size of personal space in humans. Subjects from both cohorts were recruited via online advertisement posted on the Massachusetts General Hospital Rally Website. Participants were required to be 18–55 years old and without an unstable medical or neurologic illness or diagnosis of a psychiatric disorder^41^, and have normal (or corrected to normal) vision. All experimental protocols were approved by, and all procedures were performed in accordance with the guidelines and regulation of the Mass General Brigham Healthcare Institutional Review Board. Written informed consent was obtained from all subjects prior to enrollment.

The 19 subjects enrolled in the main experiment were also screened for virtual reality (VR) sickness (vertigo or nausea) using the Simulator Sickness Questionnaire^42^ after spending ~ 10 min in the immersive VR system. No subjects were excluded due to VR sickness. Based on prior work showing that physical characteristics such as arm length may influence personal space size^32^, arm length (from the shoulder to the fingertips) and arm span (fingertips on one hand to those on the other, with horizontally oriented arms) were measured in each subject.

All data were collected prior to March 9, 2020, i.e. before the initial surge of the COVID-19 pandemic in the Boston area when the resulting infection control mandates, including social distancing recommendations, were adopted. Thus, subjects in these experiments did not wear face masks, and had no experience with mandated social distancing.

### Personal space measurement

Personal space size was measured using the well-validated Stop Distance Procedure (SDP)^11,43^. Two types of SDP measurements were collected, using a ‘passive’^44^ and an ‘active’ procedure^45^. In addition, each procedure was repeated in two different modalities: in real physical space with human intruders, and in an immersive virtual reality (VR) environment using human-like avatars. (Here we use the term ‘intruder’ to refer to the study staff member who positioned themselves at different distances from the subject or the analogous virtual character in the VR environment).

In the ‘passive’ measurements, subjects were asked to stand still, facing the intruder, who initially stood 3 m from the subject. Subjects were instructed as follows: “*Please stand still as my colleague walks slowly towards you. Say ‘okay’ when they reach a distance at which you would normally stand to talk to someone who you have just met. When you say ‘okay’, they will pause… Please make sure to maintain eye contact with my colleague throughout the procedure*”. The intruder maintained a neutral facial expression throughout the measurements. The distance between the subject and the intruder (the ‘stop distance’) was measured by a staff member after each trial, defined as the distance between the proximal tips of their shoes on the floor. The stop distances were considered to represent the preferred size of personal space for each subject.

The ‘active’ measurements began similarly, with the subject and intruder standing 3 m apart. In this procedure, the instructions were: “*Please walk up to my colleague and stop at the distance at which you would normally stand to talk with someone who you have just met. Please make sure to maintain eye contact with them throughout the procedure*”. Again, the subject was instructed to stop and say ‘okay’ at the final chosen distance. This stop distance was measured and recorded by a staff member.

**Figure 2:**
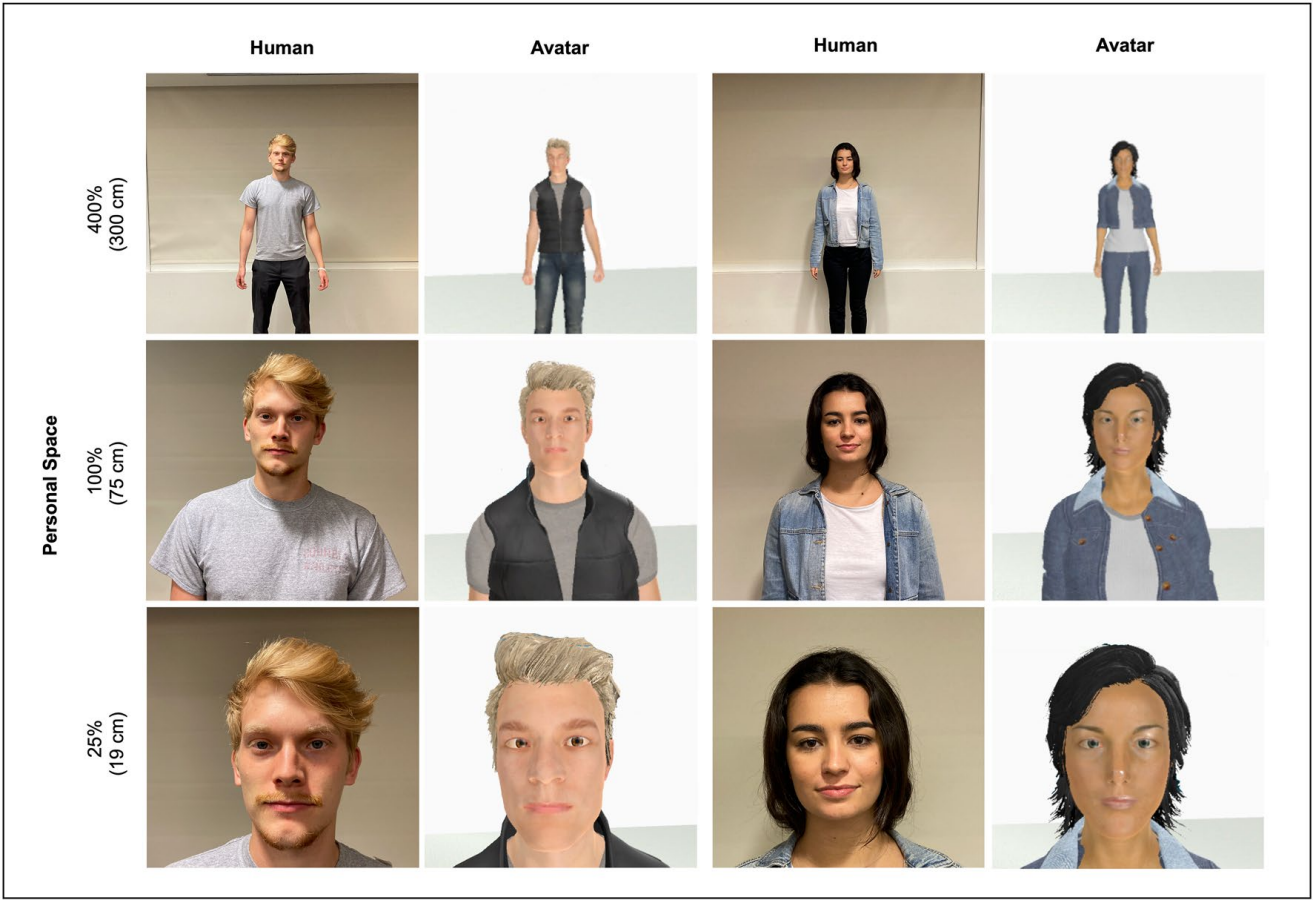
Examples of human and virtual ‘intruders’. The examples shown here include real humans (left and middle right rows, a male and female, respectively) and avatars (middle left and right rows) which served as ‘intruders’ in the Stop Distance Procedures and the Distance Range Experiments. Human and virtual intruders were positioned at systematically varied distances. The distance variations were based on a percentage of each subject’s personal space size. For this illustration, the personal space size was arbitrarily set at 75 cm. The avatars displayed here are highly similar in appearance to the human subjects shown, in order to illustrate *minimum* possible differences in appearance between the humans and avatars that could be achieved. Despite the similarities of these examples, it is easy to distinguish the avatars from the human subjects. In the actual experiment, the humans and avatars viewed by each participant were not necessarily similar in appearance. Also, additional cues made it trivial to distinguish the real versus virtual intruders (e.g., subjects were clearly aware of whether they were wearing VR goggles, or not).

These experiments measured responses to both real humans and to human-like avatars (Fig. 2; Informed consent was obtained from the two individuals shown in Fig. 2a to publish these images in an online open-access publication). The VR-based SDP procedures and instructions were generally the same as those described above, except that an avatar (who was controlled by a member of the study staff) was presented instead of a human, within an immersive environment (a generic virtual room).

In the procedures using human intruders, we presented one male and one female to each subject (both of whom were previously unknown to the subject). In the procedures using avatars, there were four different intruders (two male and two female avatars). For each subject, each type of measurement (active and passive) of the SDP was repeated twice for each human or avatar intruder, resulting in 4 trials with humans and 8 trials with avatars per SDP type. In the VR environment, the height of each avatar was set to the height of the subject, and the approach speed was set at 0.1 m/sec. In the version using humans, the human intruders were trained to walk at approximately the same speed as the avatars. Across subjects, the following were counterbalanced: (1) the presentation order of the intruders, (2) the orders of procedure modality (human or avatar), and (3) the type of SDP measurement (active or passive).

### Immersive virtual reality system

The HTC Vive Virtual Reality System included a wired head mounted display (HMD), two sensors mounted on tripods, and two handheld controllers. This HMD system displayed a neutral room within which avatars could be placed at different distances from the subjects, and then walk towards the subjects while maintaining eye contact (i.e., in the passive SDP), or to remain stationary while subjects walked towards them (i.e., in the active SDP). Personal space measurements were collected using a custom-designed program (Productive Edge; https://www.productiveedge.com/), generated on a Unity engine, running on a SteamVR platform. The virtual display was stereoscopic, with a resolution of 1080 × 1200 pixels per eye, with a 110° field of view and a refresh rate of 90 Hz.

### Distance range measurements

Based on the above measurements of personal space size (also referred to as ‘interpersonal distance’ here) collected using the active SDP procedure, 5 percentages (25%, 50%, 100%, 200% and 400%) of the subject’s average personal space size were calculated for each subject, and for each modality (averaged over the two genders). The active SDP measurements were chosen for these calculations, instead of the passive SDP values, because of the slightly greater reliability in the former measurements (see below). Then the human and avatar intruders were presented to the subjects at these five distances (real and virtual distances, respectively), in a counterbalanced, pseudorandomized order.

For each modality (human and virtual), the same procedure was conducted twice (using the same order of modality, human or virtual first), for a total of 4 presentations. During the first two presentations, skin conductance responses (SCRs) to an intruder were measured. During the second two presentations, the subjective levels of discomfort were rated by the subject. This fixed order of measurements (with SCR measurements followed by the discomfort ratings) was chosen to minimize habituation of the SCRs to the stimuli, and to avoid potentially confounding effects of the subjective ratings on the SCRs. In the first two presentations (to measure SCRs), each distance was repeated twice per intruder in each modality (real and virtual; the order of which was counterbalanced across subjects). In the second two presentations (to measure subjective ratings), each distance-by-intruder combination was presented once. The overall sequence of procedures and experimental design is shown in Supplementary Figure S1.

To both human and avatar intruders, the experimental timing was as follows: (1) eyes closed, (2) control presentation of empty room (real or virtual), (3) eyes closed, (4) experimental presentation of the intruder in the room (real or virtual), and repeat. The intertrial interval before and after the “eyes closed” conditions was systematically varied, with a mean of 5 s. During the SCR trials, the subjects were instructed to begin with their eyes closed, then to open their eyes for 5 s to a blank room, in which no intruder was present. This inter-trial interval allowed physiological responses to return to baseline between trials. Subjects were then instructed to close their eyes again, and then to open them when cued, at which time an intruder was presented at a given distance. This opening and closing of the eyes was used to avoid possible artifacts due to movement of the human intruder re-positioning themselves within the room. The subject was then instructed to stand still during the presentation of the intruder, and to maintain eye contact for a duration of 10 s, as cued by study staff. During the second (discomfort ratings) trial, subjects were also asked to open and close their eyes, without any blank room condition. When subjects opened their eyes to view the intruder, they were asked to rate their agreement to 3 statements (“I want to move away”, “I am making this person uncomfortable” and “This person is making me uncomfortable”; order counter-balanced across subjects) on a Likert scale ranging from 1 to 5 (1 = agree not at all; 5 = agree very much).

Prior to beginning the Distance Range experiment, two 11-mm Ag/AgCl sensors filled with isotonic paste were placed 14 mm apart on the hypothenar surface of the subject’s non-dominant hand. Skin conductance level (SCL) was measured (in micro siemens) using the MP150 data acquisition system (BIOPAC Systems, Inc., Goleta, CA) at a sampling rate of 2000 Hz, in analogue format, then digitally resampled to 125 Hz before analysis. An event marker was placed at the start of each stimulus trial to allow for measurement of SCL before and after stimulus presentation. SCR amplitude was calculated for each trial by subtracting the mean SCL 1 s before stimulus presentation from the peak of the SCL during the stimulus presentation. We chose to use this simple standard approach, focusing on capturing the amplitude of the response to each stimulus, corrected for possible drifts in SCL due to prior stimulus trials, because it requires no assumptions or models and has been used extensively in prior studies^46–48^.

### Statistical analyses

Pearson’s correlations were calculated to compare personal space size across SDP measurements and the two SDP modalities (real and virtual). For the Distance Range measurements, repeated measures ANOVAs were used to test for differences in discomfort ratings (conducted using the average of the ratings for the three statements at each distance, to limit the number of statistical tests) and SCRs within and across modalities and across the personal space distances. These ANOVAs were followed by post-hoc paired samples t-tests. Significance values were corrected for multiple comparisons (Bonferroni), within each comparison.

Most of the data collected in this study were normally distributed. However, several distributions were slightly skewed, such as the personal space size data collected using the active Stop Distance Procedure in the reference cohort of 30 subjects. However we chose to use parametric tests throughout, given that normality requirements can be relaxed for data with the sample sizes of this study^49–51^ and because this approach facilitates comparisons across the different portions of our data and with the findings of related studies also using parametric tests.

### Model fitting

The SCR and subjective rating data as a function of distance to intruder were modeled using three different curves, all of which were strictly decreasing functions. They include a power law function $\left(f\left(x\right)=a {x}^{-b}\right)$, an exponential function $(f(x)=a {e}^{-bx})$ and a logarithmic function $(f(x)=a-b\mathrm{ log}\left(x\right)$. In each case, $f(x)$ represents the variable of interest (SCR or subjective rating), $x$ is the distance from the intruder, and $a,b$ are pararameters (positive real numbers) to be inferred from the data, using a least square method. As an index for ‘goodness of the fit’, the normalized Root Mean Square (RMS) error was reported in each case. The RMS error was defined as $RMS=100 \sqrt{\frac{1}{n}{\sum }_{i=1}^{n}{\left(\frac{{y}_{i}-f({x}_{i})}{{ y}_{i}}\right)}^{2}}$, where ${x}_{i}$ is the $ith$ distance, ${y}_{i}$ is its corresponding $ith$ measurement (SCR or subjective rating), and $n$ is the total number of data points. The coefficient 100 was added to represent the RMS of Error as a percentage.

## Results

### Within-subject measurements of personal space

These experiments relied on extensive within-subject measurements, in order to increase the signal/noise ratio for each measurement. To test whether such repeated measurements would remain stable over time, we measured the reliability of SDP measurements of personal space size in 30 subjects, with human intruders only. In one session, only passive measurements were collected. In another session, active and passive measurements were collected. The order of passive and active measurement (and the order of intruder gender) was counterbalanced pseudo-randomly across subjects. For each SDP measurement type (passive vs. active), personal space size (i.e. the preferred interpersonal distance) was measured six times.

**Figure 3:**
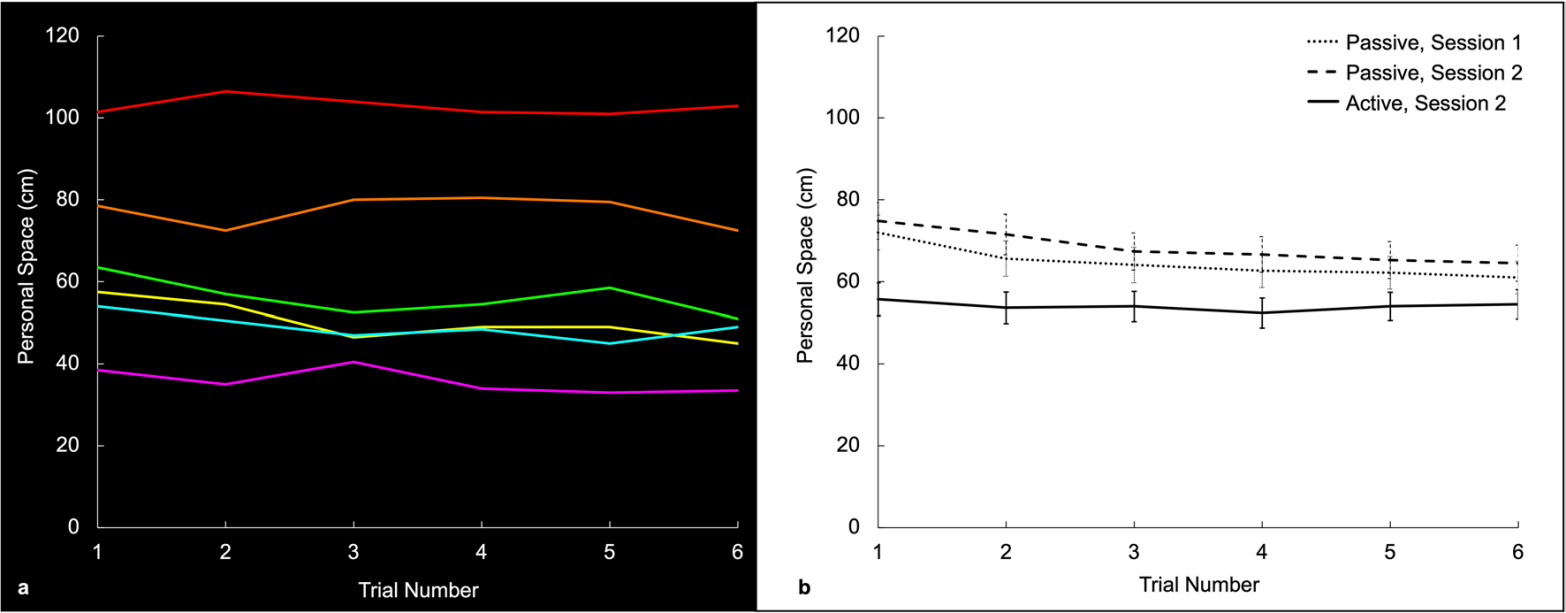
Measurements of personal space size are highly reliable within subjects, but vary across subjects. (**a**) Examples of multiple measurements of personal space size, from six subjects, using the passive Stop Distance Procedure (SDP). Data from each subject is shown in a given unique color, arbitrarily chosen for each subject. The SDP values of personal space size remained quite stable across the six trials for each individual subject. (**b**) Group-averaged personal space size (n = 30), measured using the passive SDP (see the dotted line (Session 1) and the dashed line (Session 2, occurring 2–3 days later)), and using the active version of the SDP (see the solid line; Session 2). Again, these group-averaged SDP data show that personal space size is stable over time, and measurements collected using the passive SDP are consistently larger than those collected using the active SDP. Error bars represent one standard error in each direction.

As illustrated in Fig. 3a, we found that the personal space size measured in a given subject (relative to a given human intruder) was relatively stable across all six trials, particularly for the active SDP measurements. For instance, the correlations across all active SDP measurements were high (all r > 0.929), and the average of first and last (sixth) measurements were statistically identical to each other (first distance = 55.7 cm ± 4.09; last distance = 54.5 cm ± 3.6) (Fig. 3b). Nevertheless, consistent with past studies, we observed wide variability in personal space size *across* individual subjects (e.g., mean active personal space size = 63.3 cm, s.d. = 25.2 cm). Because of the high reliability of values *within* each subject, and the considerable variability *between* subjects, we chose the SDP measurements collected using the active procedure to calculate the personal space increments for each subject used in the Distance Range experiment (see below).

In the SDP measurements collected using the passive procedure, we found that the personal space size values were slightly higher in the initial two trials, then quickly stabilized. For instance, in the average passive SDP values shown in Fig. 3b, the mean in the third trial was 10.47% less than that in the first trial. However, after stabilization (e.g. trials 4 through 6), differences across trials were not significant (all *p* > 0.112), and the correlations across those last three trials were high (all r > 0.957).

Overall, these results suggest that extensive repetition of measurements of personal space size within a given subject do not evoke progressively weaker responses. That is, we found no habituation of the response due to familiarity or diminished orienting/attentional effects, at least beyond the initial trials. These data suggest that personal space is regulated in a relatively stable manner, in response to a given intruder.

### Personal space relative to human vs. avatar intruders

Several experiments using virtual reality (VR) environments have suggested that virtual human-like ‘avatars’ can evoke responses related to personal space regulation that may be similar to such responses to real human subjects, even though such avatars are clearly distinguishable from humans^17,19,52–59^. However, the extent to which the properties of such personal space responses to avatars corresponds to those to humans, in otherwise similar contexts, remains unresolved.

**Figure 4:**
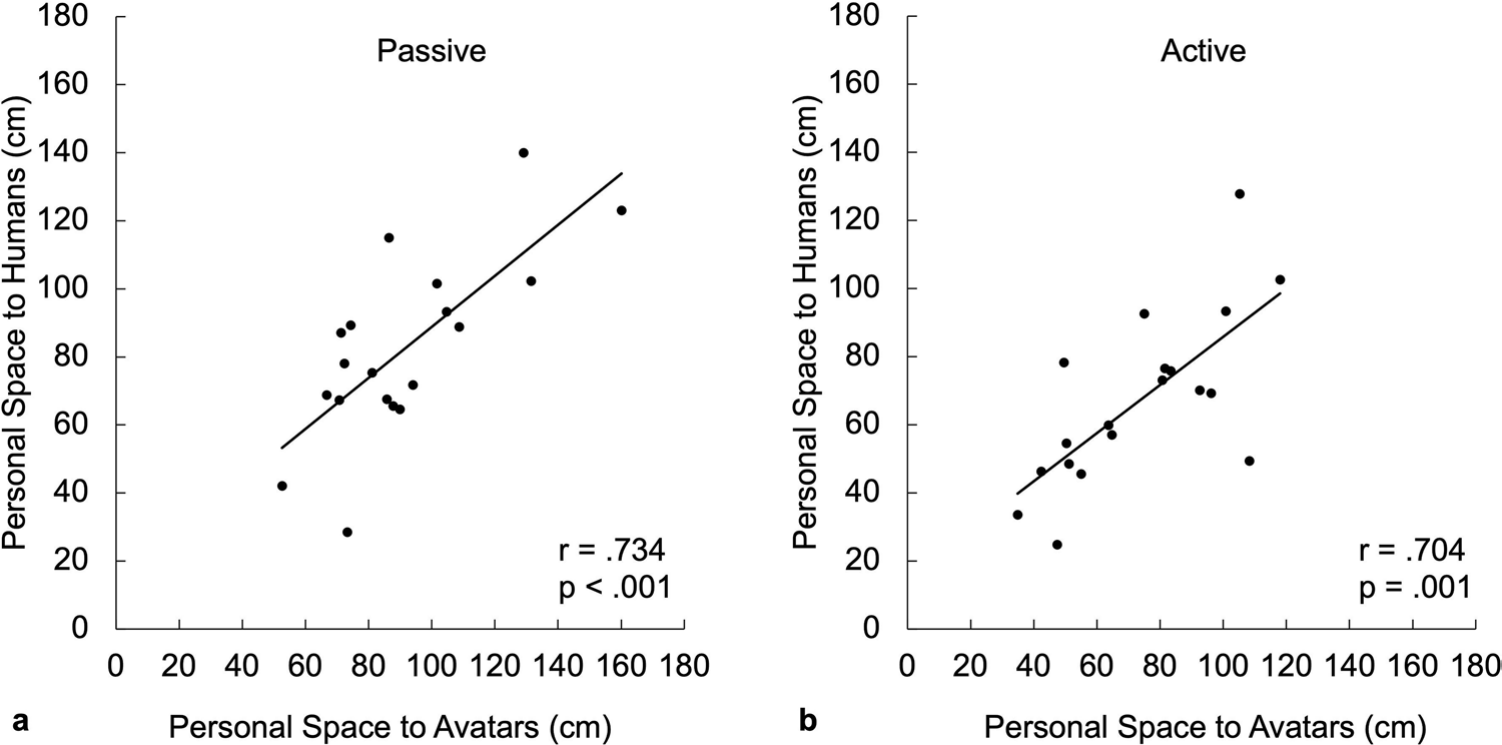
Personal space size measured to human intruders (y axis) is highly correlated with the personal space size measured to virtual intruders (x axis). Each dot represents the average personal space size measured in each subject using the Stop Distance Procedure (SDP) (n = 19). Panel (**a**) shows the correlation in the passive SDP measurements, and Panel (**b**) shows the correlation in the active SDP measurements.

Figure 4 and Supplementary Figure S2 show the relationships between the experimental SDP measurement (passive vs. active) and the experimental intruder type (human vs. avatar). For both humans and avatars, measurements of personal space size were larger when measured using the passive compared to the active SDP procedure (humans: t = 5.376, *p* < 0.001; avatars: t = 3.547, *p* = 0.002), consistent with prior work^54^ (but see^19^). Critically, the measured personal space size to avatars was highly correlated with those to humans (passive: r = 0.734, *p* < 0.001; active: r = 0.704; *p* < 0.001).

### Discomfort ratings across a range of interpersonal distances

The above data are based on measurements of personal space using the SDP, which is a threshold measurement of discomfort^11,12^. However, it is now well recognized that discomfort levels vary with distance from an intruding person, when those distances are *within* the personal space threshold defined by the SDP.

**Figure 5:**
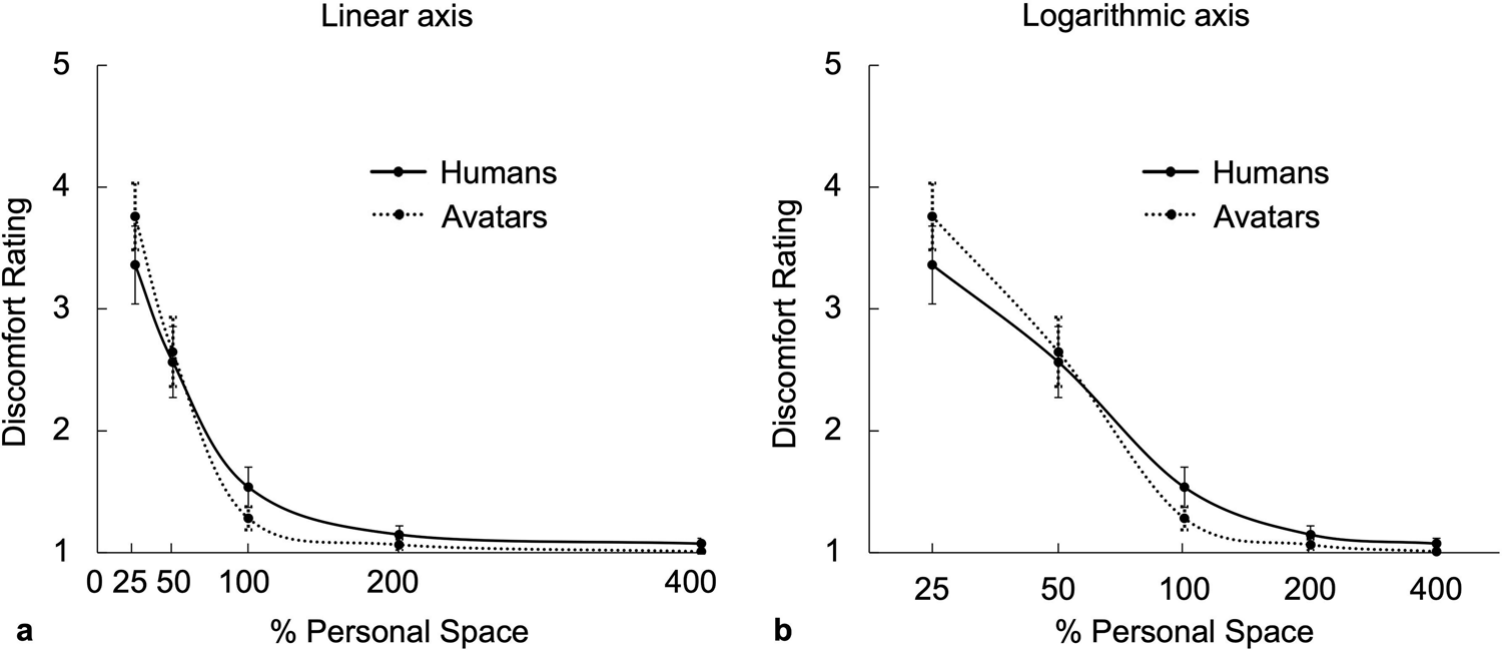
Discomfort ratings across a range of distances, normalized to individual personal space size. Group-average (n = 19) ratings of discomfort (the average of ratings of three statements, see text), on a Likert scale of agreement ranging from 1 (‘not at all’) to 5 (‘very much’), to variations in the distance (as a percentage of the personal space size of each subject) (x axis) in response to humans (solid line) and avatars (dotted line). Discomfort ratings were highest for both humans and avatars presented at the closest distance (25% of personal space size). Conversely, the discomfort ratings were lowest to both humans and avatars (at and near baseline of 1 = lowest possible ratings) when presented at distances further than the personal space boundary, i.e. 200% and 400% of a given subjects’ personal space size.

To measure variation in discomfort relative to this personal space threshold, we presented human and avatar intruders at five systematically-varied distances from the subject. These distances corresponded to 25%, 50%, 100%, 200% and 400% of the personal space size that was defined earlier in each subject, based on the active SDP procedure. For avatar-based intruders, the binocular disparity and the head size were calculated and presented to match those of real humans at a comparable distance range. During each trial, the subject was asked to rate their agreement to three specific statements to quantify their subjective level of comfort. The discomfort ratings to each question at each distance for both human and avatar intruders are shown in Supplementary Figure S3, and the average responses to the three questions are shown in Fig. 5.

Overall, we found little difference among the responses to the three questions, compared to their average, for both the humans and avatars. A repeated measures ANOVA revealed no significant main effect of SDP modality (real vs. virtual, F = 0.516, *p* = 0.482) but revealed a significant main effect of distance (F = 5.873, *p* < 0.001; see Supplementary Table S1 for additional ANOVA results). This effect was due to significantly higher discomfort ratings at the closer distances (25%, 50%) compared to the personal space boundary (100%) and beyond it (200%, 400%), in both modalities (see Supplementary Table S2 for pairwise t-test results). Responses at the ‘baseline’ values, outside of personal space (200% and 400%), did not differ significantly from each other, in either the real and virtual modalities. Lastly, a modality by distance interaction (F = 4.375, *p* = 0.003) resulted from non-significant trends towards differences between the responses to real vs. virtual humans at the 25% and 200% distances (Supplementary Table S1).

Taken together, these results are most consistent with the model shown in Fig. 1d. These results do not support a two-armed V-shaped function, which is a defining feature of the social equilibrium model; Fig. 1e)^19,27^.

### Model testing of ratings data

**Figure 6:**
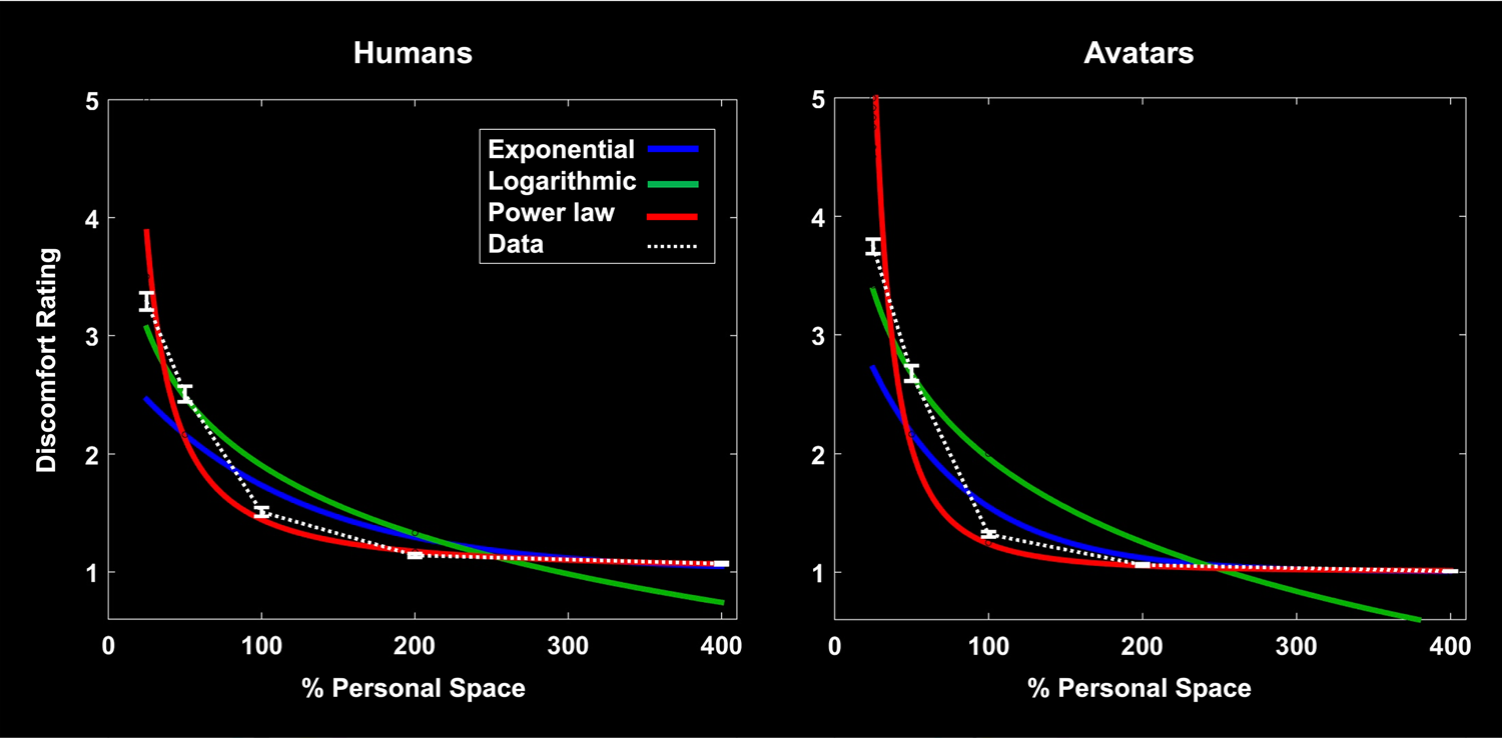
Model fitting of discomfort ratings data. Average ratings of discomfort (y axis) as a function of personal space size (in %, on the x axis) to either human (left panel) or avatar (right panel) intruders. In both panels, the data is shown as a white dotted line. Brackets indicate one standard error. Three possible functions were tested to model the average fall-off of comfort ratings, including exponential (blue), logarithmic (green), and a power law (red). The power law function produced the best fit.

To model the fall-off of this discomfort-by-distance function, we fit three different monotonically decreasing functions to the average discomfort ratings as a function of the distance between subjects and intruders. In the responses to both humans and avatars, the fall-offs were better fit by a power law function (Root Mean Square (RMS) error of the fit = 10.37% and 12.92% for humans and avatars, respectively), compared with either an exponential (RMS error 15.70% and 16.97% for humans and avatars, respectively) or a logarithmic function (RMS error 19.64% and 31.32% for humans and avatars, respectively (Fig. 6).

Moreover, to both humans and avatars, all three functions showed a superior fit to scales of *interpersonal* distance, rather than to *absolute* distance. For instance, for the power law fit, the RMS error for the absolute distance = 28.99% and 28.76% for humans and avatars, respectively, compared to the RMS error for interpersonal distance = 10.37% and 12.92% for humans and avatars, respectively. This supports the hypothesis that these discomfort responses reflect gradations in interpersonal distance (i.e. normalized to each subject), rather than absolute distance.

### Skin conductance responses across a range of interpersonal distances

It has been suggested that the discomfort evoked by personal space intrusions might manifest itself in physiological variations of the skin conductance response (SCR) at correspondingly different interpersonal distances, given that SCR is thought to reflect variations in ‘arousal'^59–65^, based on sympathetic nervous activity. Given that expectation, we also measured whether (or how closely) such SCR responses to avatars would match those to the human subjects, as a further test of our rough sketch model.

We measured SCR amplitudes to presentations of both humans and avatars, over the same distance range tested above, i.e. 25%, 50%, 100%, 200% and 400% of each subjects’ individual personal space. Following data collection, one subject was excluded as an outlier based on a priori criteria (average skin conductance responses outside of 1.5* interquartile range); thus these analyses are of data collected from the remaining 18 subjects. The stimuli were identical to those used in the rating measurements. We measured and averaged the SCRs across 4 or 8 trials per distance, relative to the real humans or avatars, respectively.

**Figure 7:**
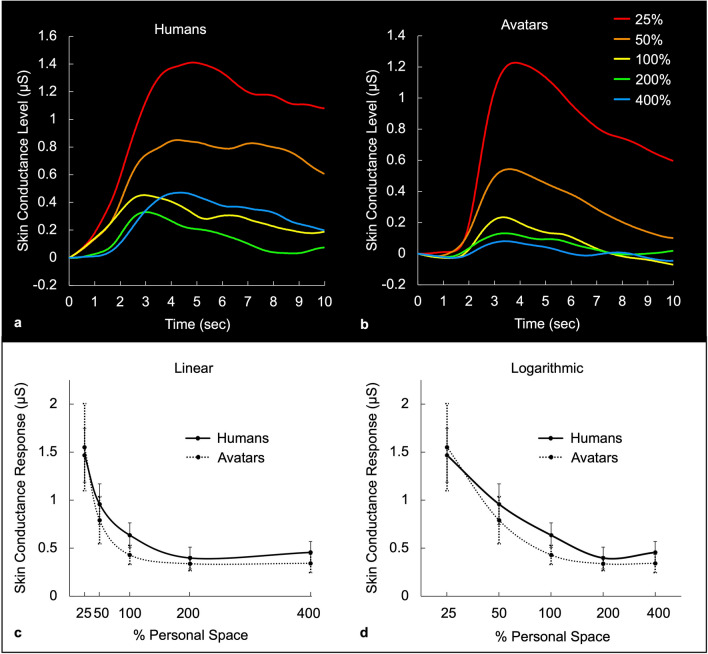
Average skin conductance responses across a range of distances, normalized to individual personal space size. The top panels show the average time courses of skin conductance levels measured in response to humans (panel **a**) and to avatars (panel **b**), when presented at different percentages of each subject’s personal space size. The bottom panels show the average peak skin conductance response amplitudes (‘arousal level’) across five percentages of personal space size (x axis), for humans (solid line) and avatars (dotted line), plotted on a linear axis (panel **c**) and on a logarithmic axis (panel **d**).

**Figure 8:**
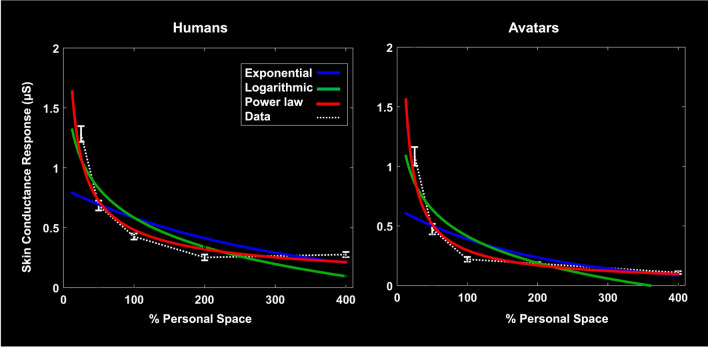
Model fitting of skin conductance responses. SCR peak responses (y axis) as a function of personal space size (in %, on the x axis) in response to either a human (left panel) or avatar (right panel) intruder. As in the ratings data, the averaged SCR response (dotted white line) was best fit by a power law (red), compared to either an exponential (blue) or a logarithmic (green) function.

We found that the SCR functions in response to humans and avatars (Figs. 7 and 8) were similar to each other, and also similar to the discomfort rating functions (Figs. 5 and 6, and Supplementary Fig. S3). Supporting this observation, a repeated measures ANOVA revealed a main effect of distance (F = 17.116, *p* < 0.001), with no main effect of modality (real vs. virtual; F = 0.146, *p* = 0.707) or modality by distance interaction (F = 0.294, *p* = 0.881). Follow-up paired sample t-tests showed no differences between SCRs elicited by humans vs. avatars at each distance (all *p* > 0.2; see Supplementary Table S1). Also, within each modality (real and virtual), significantly higher SCRs were found at the closer distances (25%, 50%) compared to the further ones (100%, 200%, 400%), and there were no significant differences between the SCRs elicited at any of the further distances (200% vs. 400%, 100% vs. 200%, and 100% vs. 400%; see Supplementary Table S3 for pairwise t-test results).

### Model testing of SCR data

As in the ratings data, we found that the fall-off in SCR values to both human and avatar intruders was well fit by a power function (RMS error 18.43% and 18.57% for humans and avatars, respectively), and less well fit by an exponential (RMS error 39.39% and 43.57% for humans and avatars, respective) or logarithmic function (RMS error 39.02% and 53.02% for humans and avatars, respectively (Fig. 8).

### Consistency across the data and effects of physical features

**Figure 9:**
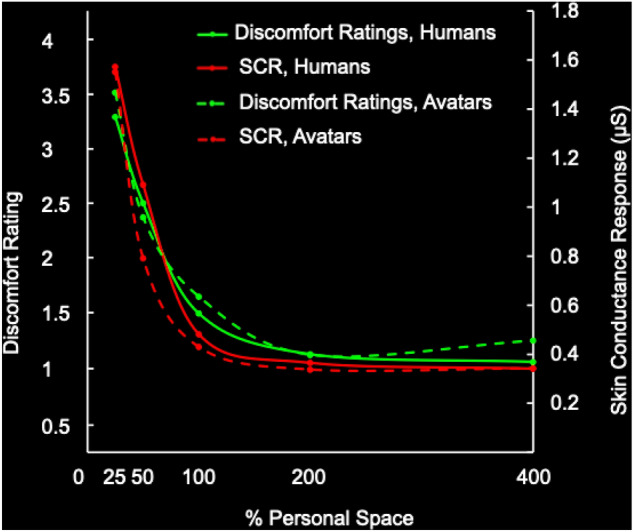
Similarity of discomfort ratings and skin conductance responses, to humans and avatars. The discomfort ratings are shown in green and the SCRs are shown in red. The ratings and SCRs to humans and avatars are displayed with solid and dashed lines, respectively. The minimum rating was 1, so the baseline values (further than the averaged personal space, e.g. at 200% and 400%) was near (but slightly above) 1. In the SCR measurements, the baseline values averaged near 0.4 microSiemens. Both the rating and SCR scales are linear. The scaling of ratings to SCR was otherwise arbitrary.

To highlight the consistency across the two measurements in the discomfort-by-distance functions, in responses to both humans and avatars, both the discomfort ratings and SCR data are shown in Fig. 9.

Lastly, we found no statistically significant correlations between measurements of arm length or span and the personal space (SDP) measurements, the discomfort ratings or the SCR data.

## Discussion

Overall, this study found little or no significant differences between the interpersonal distances measured in response to real and virtual human intruders. This similarity in personal space with respect to humans and avatars was confirmed in extensive measurements of rated discomfort levels across a range of interpersonal distances, including those closer and further than each subject’s personal space boundary. This decrease in comfort at distances closer than the personal space boundary (i.e. the ‘fall off’ in discomfort at increasing distances) was well fit by a power law, for the responses to both humans and avatars, and for both psychological (ratings) and physiological (SCR) measurements.

These results suggest that responses to simulations of humans (avatars) at different virtual distances share neural mechanisms with those involved in responding to real humans at corresponding actual interpersonal distances. Such evidence for shared neural processing supports the hypothesis that the brain performs a ‘rough sketch’ of personal space at an early stage of information processing.

### Variations in discomfort across interpersonal distance

The classic stop distance measurements of personal space described above define a (binary) *threshold* response, i.e. a specific distance at which the subject begins to feel uncomfortable in response to an intruder. At distances that are progressively closer than that threshold-defined personal space size, many prior studies (and the data here) show that the level of discomfort increases accordingly. Prior studies have reported that this gradient is linear with personal distance (with strong non-linear contributions)^24^, or logarithmic^59^, or one arm of a wider ‘V’ shaped function^19,27^.

Here, the fall-off was well fit by a simple power law, in both discomfort ratings and skin conductance data. Accordingly: (1) the highest responses occurred at the closest distance tested (25% of personal space); (2) the lowest (baseline) responses occurred at the furthest distances tested (200 and 400%); (3) the function approached baseline near the behaviorally-defined personal space boundary (100%); and (4) intermediate responses were found at an intermediate distance (50%).

Stevens^66^ formulated the quantitative relationship between the perceived magnitude of a stimulus and its physical intensity as a power law. That relationship became known as a “fundamental law of psychophysics”^67^, which has been confirmed empirically in multiple sensory systems and tasks. For instance, in the visual system, a power law function has been attributed to the relationship between stimulus luminance and brightness, the physical size of an stimulus and its perceived size, the purity of a hue and its saturation, and several other attributes of visual stimuli and their perceived magnitude^68^. Physiologically, power law relationships have been demonstrated in neural activity across different scales, ranging from the firing rate of single neurons in visual cortex in response to stimuli with different intensities, the number of neurons activated by stimuli with different intensity levels^69^, the relationship between luminance and visual-evoked potential latency^70^, and even to the relationship between cognitive complexity (numerosity) and neural activity in the prefrontal cortex of monkeys^71^.

The mechanistic roots of the power law relationship can be inferred from the activity of the neuronal substrate at various levels. At the level of a single neuron, a noisy linear-threshold function can account for the power law relationship between the input voltage and the firing rate of a neuron^72^. At the level of neural circuits, a power law nonlinearity can arise from an interaction of neurons with nonlinear sigmoid activation functions through a feedback loop^73^. At the level of neural ensembles, power laws can arise from averaging multiple independent exponential decay processes^74^. More generally, power laws, being quite common in biological systems^75^, can arise from the properties of self-organized critical systems that are far from thermodynamic equilibrium^76^.

Thus, power laws are common in models of sensory systems, and the evidence here for power law responses to personal space intrusions is broadly consistent with our rough sketch model of personal space, at early (e.g. more sensory-dominated) stages of computation.

It is also broadly consistent with evidence from fMRI studies for increases in activity in areas of parietal cortex and closely connected brain regions in response to images of human faces that are located within, but not outside of, virtual personal space^77^; the amplitude of this response is proportional to the proximity of the stimulus. Additional work can reveal whether such fMRI responses also follow a power law function.

### Within-subject and between-subject variability in personal space

It is well established that SDP measurements based on *absolute* distance vary significantly between individuals^11^. To account for this variation, Hayduk^24^ argued that it is helpful to normalize (or ‘proportion’) personal space measurements across individuals. Consistent with this, we found that such a normalization yielded data that was more consistent across individuals, and less noisy, compared to variations in absolute distance. This scaling of *interpersonal* vs. *absolute* space implies that a specific set of brain mechanisms are engaged to calculate and regulate personal space in all subjects, but that the values in a given subject scale by a constant.

In contrast to the wide variation *between* individuals, we found high reliability *within* subjects, in repeated measurements of stop distance, with a mean inter-trial reliability of *r* = 0.879 (lowest correlation of 0.763) among all six trials of passive and six trials of the active SDP measurements. This high reliability is consistent with prior studies, which reported an inter-trial reliability of *r* = 0.81^24,78^. Although the measurements of the passive SDP task showed slightly higher initial values, the small increase in the passive mean SDP scores may arise because in the passive (but not the active) condition, subjects *anticipated* an impending personal space intrusion, which could be avoided only by saying ‘stop’. Thus, slightly earlier ‘stop’ commands resulted in a slightly larger personal space size, perhaps reflecting a more general contribution from threat detection mechanisms, in addition to basic mechanisms regulating personal space. Regardless, the near-constant values evident in the *active* condition, and after stabilization in the passive version, suggest that repeated conventional measurements of personal space size reflect surprisingly little or no habituation. Other studies have also reported that values of personal space size are highly replicable over time, over durations ranging from 1 to 60 s^79^, to multiple weeks between measurements^80^. Based on these data, Hayduk^78^ concluded that “there are few social science phenomena that can be measured as precisely as personal space”.

Here, the high reliability of personal space measurements was important for two reasons. First, this property was crucial in order to reduce experimental noise by extensive signal-averaging, based on the Central Limit theorem. Second, the high reliability also supports our hypothesis that lower stage (near-sensory) brain sites calculate a rough sketch at an early stage of the personal space calculation. In visual cortex, many responses are relatively stereotyped over numerous repetitions, whereas activity of higher stage, cognitive areas is more likely to habituate.

### Alternative models of personal space

Essentially all models of personal space posit a zone immediately surrounding the subject into which intrusion by another person evokes discomfort (see Fig. 1). In addition, a ‘social equilibrium’ model’^27^ further proposed a second zone of increasing discomfort in response to persons that are located further (rather than nearer) than the conventionally-defined personal space boundary^25,26^ (Fig. 1e). This hypothetical second zone of increased discomfort has been termed an ‘extrusion’ zone^19,24^. A recent study reported experimental evidence for such a discomfort-producing extrusion zone, based on higher ratings of discomfort found both within and outside of the personal space boundary^19^. Here, we did not find evidence for such an extrusion zone, even though our measurements spanned a larger range of absolute distances than this prior study. Perhaps differences in contextual factors or task instructions account for the presence or absence of an extrusion effect. For example, if discomfort ratings in prior studies reflected responses to violations of social norms for interpersonal distances, subjects may have reported some subjective discomfort at distances outside of their expected personal space boundary.

This unresolved question bears specifically on our understanding of experiences of social distancing during the COVID-19 pandemic. The existence of an extrusion zone would predict that standing at pandemic-related social distances (e.g. 2 m) would induce additional discomfort, compared to standing at typical interpersonal distances^81^. However if there is no extrusion zone, no increase in discomfort would occur during social distancing.

### A rough sketch of interpersonal distance

Based on the results described here, we propose that the neural calculation of personal space includes an imprecise initial value of personal space size (i.e. a rough sketch) at an early stage of spatial encoding. Single-neuron recording in monkeys^38,40,82^, behavioral studies^39^, neuropsychology^40,83–86^, and fMRI results in humans^35,37,87^ suggest that such an initial stage of personal space computation might involve parietal cortex. Our general hypothesis is that the *eye-centered* spatial encoding in visual cortex is integrated into a broader body*-centered or ego-centered)* spatial mapping in/near inferior parietal cortex. Such a body- (or person-) centered coordinate system could be used as an initial substrate for mapping personal (as well as non-social) space.

An implied corollary of this hypothesis is that this rough sketch is communicated to higher stage cortical areas which fine-tune that initial value of interpersonal distance, based on more subtle cues (e.g. whether the intruding object is a real human, and of which gender, whether presenting in an aggressive or welcoming stance, etc.). By design, our experiment focused on testing for the proposed initial rough estimate of personal space size; thus we deliberately reduced variations in higher stage variables.

Our model is broadly analogous to the ‘2 ½ D’ model of visual cortical processing, as proposed by Marr and colleagues^88,89^. In that model, lower level visual cortical mechanisms first generate an approximation of visual objects (a “2 ½ D” sketch), which is then further modified at higher computational (or brain) stages to yield the more detailed images which we ultimately ‘see’ in 3D. Related models also proposed that visual cortical inputs are processed more grossly (roughly) at lower levels, then more specifically at progressively higher levels of visual cortex^90–94^.

This ‘rough sketch’ model may also apply to other systems. For instance, in one form of the “rubber arm illusion”, a person’s real arm is hidden, and a slightly displaced artificial arm and fingers (generated using either rubber prosthetics, or programmed in virtual VR) is presented, to generate feelings of ‘ownership’ of that artificial arm, including protective responses to it^95–101^. Typically, the artificial arm can be easily discriminated from the subject’s real arm by direct inspection (since it is either an imperfect rubberized dummy or a slightly blurry, pixelated image viewed through VR goggles). Nevertheless, analogous to the results reported here, a subject’s perception can still be fooled by the approximate representation of the arm, eliciting responses ‘as if’ it were a real arm.

Another example of this effect is seen in studies using experimental achromatic images of human faces mimicking specific emotional expressions, that aim to generate responses akin to the corresponding emotions in the viewer^102–106^. Again in this case, the subject typically correctly perceives the emotional faces as simulated rather than real—but nonetheless experiences automatic, affective responses to them.

### Necessary and sufficient information for eliciting a ‘Rough Sketch’ response

Several prior studies using VR have tested for related properties of personal space regulation with respect to avatars and humans, as in the current study. However, some of these studies have reported differences between interpersonal distances (stop distance values) measured to humans when compared to those measured to inanimate human-like intruders, including plastic mannequins^17^, robots^54^, or more rudimentary avatars^55,107^. Additional experiments will be required to define the specific features of human-like avatars which must be present to evoke a need for personal space.

Link to Data Repository: https://osf.io/nq69b/?view_only=df24e799f0e84eba8064d49eb8785cfd.

## Supplementary Information

Supplementary Information.
