# Supplementary material for: Psychological and physiological evidence for an initial ‘Rough Sketch’ calculation of personal space
Source: Sci Rep. 2021 Oct 25;11:20960. doi: 10.1038/s41598-021-99578-1 (PMC8545955; doi:10.1038/s41598-021-99578-1)
Supplement: Supplementary file 1 — Supplementary Information. [file 41598_2021_99578_MOESM1_ESM.pdf]

Supplementary Materials for Tootell et al "Psychological and Physiological Evidence for an Initial 'Rough Sketch' Calculation of Personal Space"

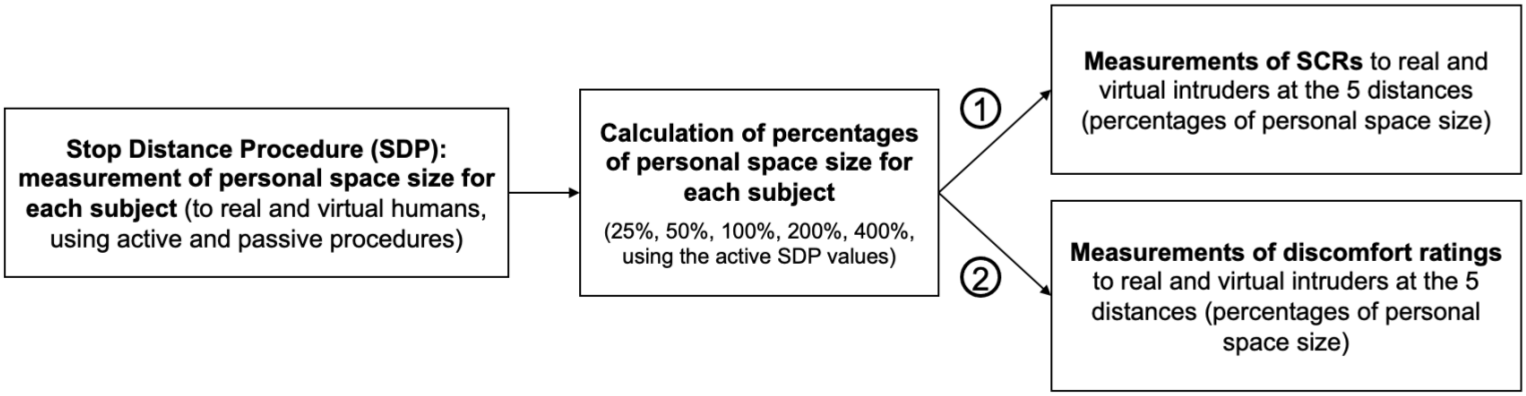

Supplementary Figure S1. Schematic diagram of the overall experimental design. This diagram shows the procedures of this study in the order that they were conducted. First, the size of each individual subject's personal space was measured using the classic Stop Distance Procedure (SDP), using both the passive and active procedures (see Methods), with respect to both real humans and avatars (males and females). The order (passive and active procedures, real and virtual modalities, male and female intruders) was counterbalanced across subjects. Following the SDP, personal space size for each subject, as measured using the active SDP procedure, in response to both humans and avatars, was calculated as well as four percentages of these two values (25%, 50%, 200%, 400%). These distances were then used in the subsequent two Distance Range Measurements, in which skin conductance responses and discomfort ratings at the 5 interpersonal distances (presented in an pseudorandomized order) were measured. The measurement of SCRs always preceded the measurement of discomfort ratings, in order to minimize habituation of the SCRs.

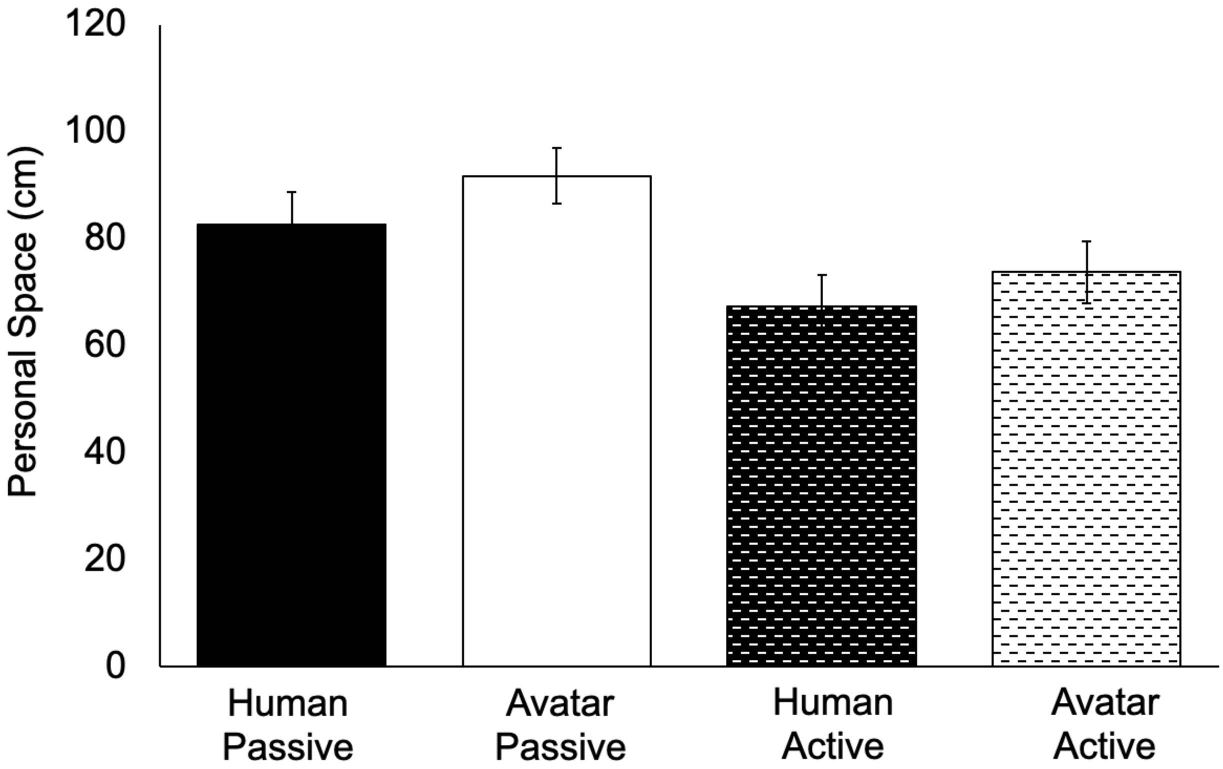

Supplementary Figure S2. Average personal space size measurements in this sample. Average personal space size to avatars (white, and white with black dashes) and humans (black, and black with white dashes, for the passive (solid) and active (dashed) Stop Distance Procedure (SDP) measurements (n=19). The passive SDP measurements were significantly greater than the active SDP measurements, as expected.

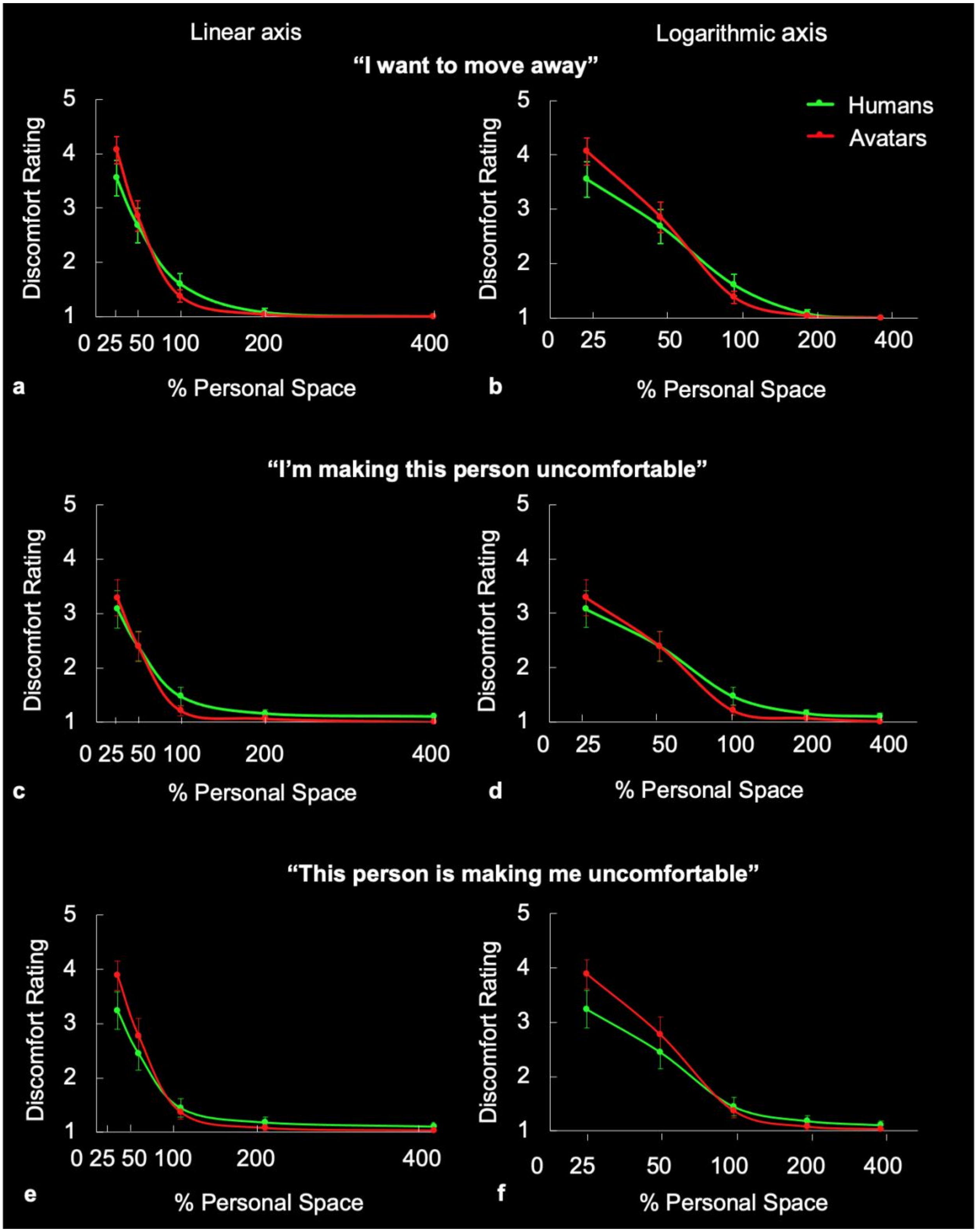

Supplementary Figure S3. Three independent discomfort ratings across a range of personal space sizes. Discomfort ratings (endorsements of three statements), plotted on either linear or logarithmic axes (left and right columns, respectively) are shown. Levels of agreement with the following statements are plotted in panels **a** and **b**: “I want to move away; **c** and **d**: “I’m making this person uncomfortable”; and **e** and **f**: “This person is making me uncomfortable”. See Figure 5 for the plots of the average ratings across all three statements.

Human vs. Avatar

a **Q1 – 'I want to move away'**

	F statistic	p-value
Modality	0.901	0.355
Distance	65.854	< 0.001
Modality * Distance	3.609	0.01

Distance	t statistic	p-value
25%	-2.601	0.018
50%	-0.723	0.479
100%	1.361	0.19
200%	0.718	0.482
400%	-	-

b **Q2 – 'This person is making me uncomfortable'**

	F statistic	p-value
Modality	0.251	0.622
Distance	36.033	< 0.001
Modality * Distance	1.549	0.197

Distance	t statistic	p-value
25%	-1.021	0.321
50%	0	1
100%	1.977	0.064
200%	2.111	0.049
400%	1.714	0.104

c **Q3 – 'I am making this person uncomfortable'**

	F statistic	p-value
Modality	3.302	0.086
Distance	51.129	< 0.001
Modality * Distance	5.926	< 0.001

Distance	t statistic	p-value
25%	-3.473	0.003
50%	-1.492	0.153
100%	0.697	0.494
200%	1.287	0.215
400%	1.372	0.187

d **Average of Q1, Q2 and Q3**

	F statistic	p-value
Modality	0.516	0.482
Distance	5.873	< 0.001
Modality * Distance	4.375	0.003

Distance	t statistic	p-value
25%	-2.783	0.012
50%	-0.749	0.464
100%	1.58	0.132
200%	1.924	0.07
400%	1.587	0.13

e **Skin Conductance Responses**

	F statistic	p-value
Modality	0.146	0.707
Distance	17.116	< 0.001
Modality * Distance	0.294	0.881

Distance	t statistic	p-value
25%	-0.156	0.878
50%	0.535	0.6
100%	1.326	0.202
200%	0.416	0.683
400%	0.629	0.538

Supplementary Table S1. Results of repeated measures ANOVAs (Modality*Distance), and between-modality comparisons (t-tests) at each distance, for the discomfort ratings and skin conductance responses. Results are listed of the ANOVA of: the discomfort ratings to the statements 'I want to move away' (**a**), 'This person is making me uncomfortable' (**b**), 'I am making this person uncomfortable' (**c**), the average ratings across all three statements (**d**) and the skin conductance responses (SCR) (**e**).

Human | **Avatar**

a Q1 – 'I want to move away'

	t statistic	p-value
25% vs. 50%	5.232	< 0.001
25% vs. 100%	6.874	< 0.001
25% vs. 200%	7.497	< 0.001
25% vs. 400%	7.819	< 0.001
50% vs. 100%	4.02	0.001
50% vs. 200%	4.962	< 0.001
50% vs. 400%	5.295	< 0.001
100% vs. 200%	2.904	0.009
100% vs. 400%	3.07	0.007
200% vs. 400%	1	0.331

b Q1 – 'I want to move away'

	t statistic	p-value
25% vs. 50%	6.665	< 0.001
25% vs. 100%	11.547	< 0.001
25% vs. 200%	12.327	< 0.001
25% vs. 400%	12.233	< 0.001
50% vs. 100%	6.115	< 0.001
50% vs. 200%	6.572	< 0.001
50% vs. 400%	6.568	< 0.001
100% vs. 200%	3.564	0.002
100% vs. 400%	3.455	0.003
200% vs. 400%	1.372	0.187

c Q2 – 'This person is making me uncomfortable'

	t statistic	p-value
25% vs. 50%	5.121	< 0.001
25% vs. 100%	5.307	< 0.001
25% vs. 200%	5.643	< 0.001
25% vs. 400%	5.903	< 0.001
50% vs. 100%	4.453	< 0.001
50% vs. 200%	4.604	< 0.001
50% vs. 400%	4.903	< 0.001
100% vs. 200%	2.191	0.042
100% vs. 400%	2.59	0.018
200% vs. 400%	1	0.331

d Q2 – 'This person is making me uncomfortable'

	t statistic	p-value
25% vs. 50%	5.127	< 0.001
25% vs. 100%	6.396	< 0.001
25% vs. 200%	6.318	< 0.001
25% vs. 400%	6.865	< 0.001
50% vs. 100%	4.698	< 0.001
50% vs. 200%	4.729	< 0.001
50% vs. 400%	5.22	< 0.001
100% vs. 200%	1.64	0.118
100% vs. 400%	2.334	0.031
200% vs. 400%	1.229	0.235

e Q3 – 'I am making this person uncomfortable'

	t statistic	p-value
25% vs. 50%	4.581	< 0.001
25% vs. 100%	6.026	< 0.001
25% vs. 200%	6.245	< 0.001
25% vs. 400%	6.36	< 0.001
50% vs. 100%	4.421	< 0.001
50% vs. 200%	4.48	< 0.001
50% vs. 400%	4.688	< 0.001
100% vs. 200%	2.041	0.056
100% vs. 400%	2.387	0.028
200% vs. 400%	1.837	0.083

f Q3 – 'I am making this person uncomfortable'

	t statistic	p-value
25% vs. 50%	5.529	< 0.001
25% vs. 100%	9.896	< 0.001
25% vs. 200%	10.591	< 0.001
25% vs. 400%	10.605	< 0.001
50% vs. 100%	5.141	< 0.001
50% vs. 200%	5.598	< 0.001
50% vs. 400%	5.634	< 0.001
100% vs. 200%	3.284	0.004
100% vs. 400%	3.059	0.007
200% vs. 400%	1.455	0.163

Supplementary Table S2. Pairwise comparisons of discomfort ratings for the five distances (percentages of personal space size). Results of pairwise t-tests comparing the ratings at each the five distances (% of personal space size of each subject) to each other are listed for the three statements: 'I want to move away' to humans (**a**) and avatars (**b**); 'This person is making me uncomfortable' to humans (**c**) and avatars (**d**); 'I am making this person uncomfortable' to humans (**e**) and avatars (**f**).

a **Human**

Skin Conductance Responses

	t statistic	p-value
25% vs. 50%	2.356	0.031
25% vs. 100%	4.191	0.001
25% vs. 200%	4.53	< 0.001
25% vs. 400%	3.814	0.001
50% vs. 100%	2.439	0.026
50% vs. 200%	3.92	0.001
50% vs. 400%	3.485	0.003
100% vs. 200%	2.702	0.015
100% vs. 400%	1.657	0.116
200% vs. 400%	-0.52	0.61

b **Avatar**

Skin Conductance Responses

	t statistic	p-value
25% vs. 50%	3.004	0.008
25% vs. 100%	2.929	0.009
25% vs. 200%	2.898	0.01
25% vs. 400%	3.099	0.007
50% vs. 100%	2.177	0.044
50% vs. 200%	2.142	0.047
50% vs. 400%	2.487	0.024
100% vs. 200%	1.207	0.244
100% vs. 400%	1.058	0.305
200% vs. 400%	-0.076	0.94

Supplementary Table S3. Pairwise comparisons of skin conductance responses for the five distances (percentages of personal space size). Results of pairwise t-tests comparing the skin conductance responses at each the five distances (% of personal space size of each subject) to each other are listed for the responses to humans (**a**) and avatars (**b**).
